# Microsleep versus Sleep Onset Latency during Maintenance Wakefulness Tests: Which One Is the Best Marker of Sleepiness?

**DOI:** 10.3390/clockssleep3020016

**Published:** 2021-04-30

**Authors:** Ludivine Des Champs de Boishebert, Pierre Pradat, Hélène Bastuji, François Ricordeau, Frédéric Gormand, Pierre Le Cam, Emeric Stauffer, Thierry Petitjean, Laure Peter-Derex

**Affiliations:** 1 Center for Sleep Medicine and Respiratory Diseases, Hospices Civils de Lyon, 69004 Lyon, France; ludivinedcdb@gmail.com (L.D.C.d.B.); helene.bastuji@chu-lyon.fr (H.B.); francois.ricordeau@chu-lyon.fr (F.R.); frederic.gormand@chu-lyon.fr (F.G.); pierre.le-cam@chu-lyon.fr (P.L.C.); emeric.stauffer@chu-lyon.fr (E.S.); thierry.petitjean@chu-lyon.fr (T.P.); 2 Center for Clinical Research, Hospices Civils de Lyon, 69004 Lyon, France; pierre.pradat@univ-lyon1.fr; 3 Lyon Neuroscience Research Center, CNRS UMR 5292, INSERM U1028, 69000 Lyon, France; 4 Laboratoire Interuniversitaire de Biologie de la Motricité (LIBM) EA7424, Team ‘Biologie vasculaire et du globule rouge’, Lyon 1 University, 69000 Lyon, France; 5 Faculty of Medicine Lyon Sud-Charles Mérieux, Lyon 1 University, 69000 Lyon, France

**Keywords:** polysomnography, sleep latency, sleepiness, wakefulness, Maintenance Wakefulness Test

## Abstract

The interpretation of the Maintenance Wakefulness Test (MWT) relies on sleep onset detection. However, microsleeps (MSs), i.e., brief periods of sleep intrusion during wakefulness, may occur before sleep onset. We assessed the prevalence of MSs during the MWT and their contribution to the diagnosis of residual sleepiness in patients treated for obstructive sleep apnea (OSA) or hypersomnia. The MWT of 98 patients (89 OSA, 82.6% male) were analyzed for MS scoring. Polysomnography parameters and clinical data were collected. The diagnostic value for detecting sleepiness (Epworth Sleepiness Scale > 10) of sleep onset latency (SOL) and of the first MS latency (MSL) was assessed by the area under the receiver operating characteristic (ROC) curve (AUC, 95% CI). At least one MS was observed in 62.2% of patients. MSL was positively correlated with SOL (r = 0.72, *p* < 0.0001) but not with subjective scales, clinical variables, or polysomnography parameters. The use of SOL or MSL did not influence the diagnostic performance of the MWT for subjective sleepiness assessment (AUC = 0.66 95% CI (0.56, 0.77) versus 0.63 95% CI (0.51, 0.74)). MSs are frequent during MWTs performed in patients treated for sleep disorders, even in the absence of subjective sleepiness, and may represent physiological markers of the wake-to-sleep transition.

## 1. Introduction

Excessive daytime sleepiness affects more than 5% of the general population [1]. Its consequences involve both the academic/professional area, with impaired cognitive performances [2], and the risk of accidents, especially on the road [3]. A recent meta-analysis reported an increased risk of motor vehicle accidents (pooled OR 2.51 (95% CI 1.87, 3.39)) associated with sleepiness at the wheel [4]. Among sleep disorders, obstructive sleep apnea syndrome (OSA) and hypersomnia significantly increase the risk of accidents, which highlights the importance of sleepiness assessment in patients suffering from these disorders [5,6,7,8].

Sleepiness evaluation is based on several tools [9]. Questionnaires are widely used as they are simple to administrate, but may be biased by sleepiness misperception or misdeclaration [10]. Among objective tests commonly used in practice, the Multiple Sleep Latency Test (MSLT) assesses the propensity to fall asleep in a quiet situation for scheduled naps [11], and the Maintenance Wakefulness Test (MWT) measures the ability to stay awake in a passive condition [12]. The MWT is considered as the gold standard in many countries for the assessment of residual sleepiness in professional drivers treated for sleep disorders. However, the MSLT and the MWT have been criticized for their non-ecological nature, far removed from driving which is a complex psychomotor task [13]. Their correlation with subjective tests is low [14,15], their validation in different populations of patients with sleep disorders is weak [16], and their validity for predicting accidental risk is limited [17]. Furthermore, although normative values have been published in healthy populations [18,19,20], they widely overlap between controls and patients [21], and the mean latency value associated with optimal driving ability is still debated. Performance on driving-simulation tests is lower in OSA patients with a mean MWT latency below 19 min [22,23], but only a latency above 33 min seems to be associated with normal driving performance, which is much higher than the normative values used in many laboratories (i.e., in France, the medicolegal threshold is 19 min) [18,24]. Finally, sleep onset latency (SOL) in MWT is defined as the time from lights out to the first epoch of sleep, i.e., more than 15 s of cumulative sleep over a 30 s epoch, whereas a few seconds of inattention or drowsiness might be fatal in a high-speed driving situation. The meaning of microsleeps (MSs) and the relevance of including them in MWT interpretation in the context of driving ability evaluation is therefore questionable.

MSs refer to short periods of sleep intrusion during wakefulness. Their polysomnography definition includes several electro-encephalography (EEG) changes (disappearance of alpha rhythm, appearance of theta waves, or even sleep features such as vertex waves or spindles), lasting from 3 to 15 s, with no blink on the electro-oculogram (EOG) nor on the frontal leads of the EEG, and sometimes associated with slow eye movements [25]. They are associated with cognitive and psychomotor lapses as assessed, for example, by a simulator driving task [26,27] and may occur altogether with motor manifestations (fall of the head, eyelids) [28]. MS scoring would improve the sensitivity of the MSLT for the diagnosis of excessive daytime sleepiness [29,30], but these findings are not unequivocal [31] and they have been poorly investigated in the context of MWT until recently. Thus, MSs (especially a series of MSs) during the MWT have been shown to predict sleep onset in patients with excessive daytime sleepiness and in untreated patients with OSA [32,33]. However, the prevalence of MSs and the relevance of their scoring in the context of residual sleepiness assessment remains unknown, whereas in clinical practice, MWTs are usually performed in order to evaluate the efficacy of treatment before enabling professional drivers to resume activity. Moreover, their association with subjective vigilance scales and with the objective sleep parameters of the polysomnography (PSG) preceding the MWT has not been explored. The objectives of our study were: (i) to assess the prevalence of MSs during MWTs performed in clinical practice for residual sleepiness evaluation; (ii) to describe MS characteristics in this context; (iii) to investigate the relationship between MS latency and various demographic, polysomnographic, and subjective questionnaire variables; and (iv) to evaluate the relevance of MS scoring during MWTs to assess residual sleepiness, after treatment, in patients suffering from OSA or hypersomnia. In particular, we compared the first MS latency (MSL) and the SOL diagnostic values for predicting subjective sleepiness as assessed by the Epworth Sleepiness Scale. Our hypotheses were that MSs would be observed with a higher prevalence and with lower latency than falling asleep, and that taking MSs into account would improve the diagnostic performance of the MWT.

## 2. Result

### 2.1. Population

Ninety-eight patients were included (82.6% male, mean (±SD) age 45.3 (±10.8) years). Demographic and clinical characteristics are depicted in Table 1. Among these 98 patients, 89 suffered from OSA (associated with idiopathic hypersomnia or narcolepsy in four) and 9 had narcolepsy alone. MWTs were performed for professional reasons in most patients (before resuming professional driving in 66.3% and before resuming a safety position in 12.2%). In 5.1% of patients, the tests were performed before the driving license test, and in 16.3% of cases, MWTs followed personal requests from patients who wished to assess their vigilance before driving. Up to 86.7% of patients were treated (continuous positive air pressure (C-PAP): 71.4%; oral appliance therapy (OAT): 3.1%; wake-promoting drugs (methylphenidate, modafinil, or pitolisant): 10.2%; combination of CPAP and wake-promoting drugs: 2.0%). A history of a public road accident that was related to excessive daytime sleepiness in the last 2 years prior to hospitalization was reported by 20.7% of patients (N = 19), without details about treatment status at the time of the accident.

### 2.2. Results of Questionnaires and Polysomnography Recordings

The Epworth Sleepiness Scale (ESS) was normal in 66.3% of patients and the Observation and Interview-based Diurnal Sleepiness Inventory (ODSI) score was normal in 57.5%. The fatigue score was normal in 92.1% of patients and 97.7% of patients had a normal Beck Depression Inventory (BDI). These results are presented in Table 1 and the PSG data are depicted in Table 2. Total sleep time was normal on average (404 min (± 69.1)) as were the amounts of N3 and REM sleep, but sleep efficiency was decreased and the arousal index was increased as compared to normative values [34]. The median apnea-hypopnea index (AHI) was within values associated with mild sleep apnea syndrome (8.2 (3.1–18.0)); 35% of patients had no OSA anymore, 31% had mild OSA, and 34% still had moderate or severe OSA despite treatment. Very few patients had nocturnal desaturation, showing overall good control of the sleep-related breathing disorder under C-PAP or OAT. On the St Mary’s Hospital Morning Rating Scale, 56.3% of patients felt that their sleep had been of fairly good to very good quality and 69.6% of them reported that their minds were fairly clear to very alert; however, 25% of patients reported that they had slept very poorly to poorly.

### 2.3. MSs Description

**Figure 1:**
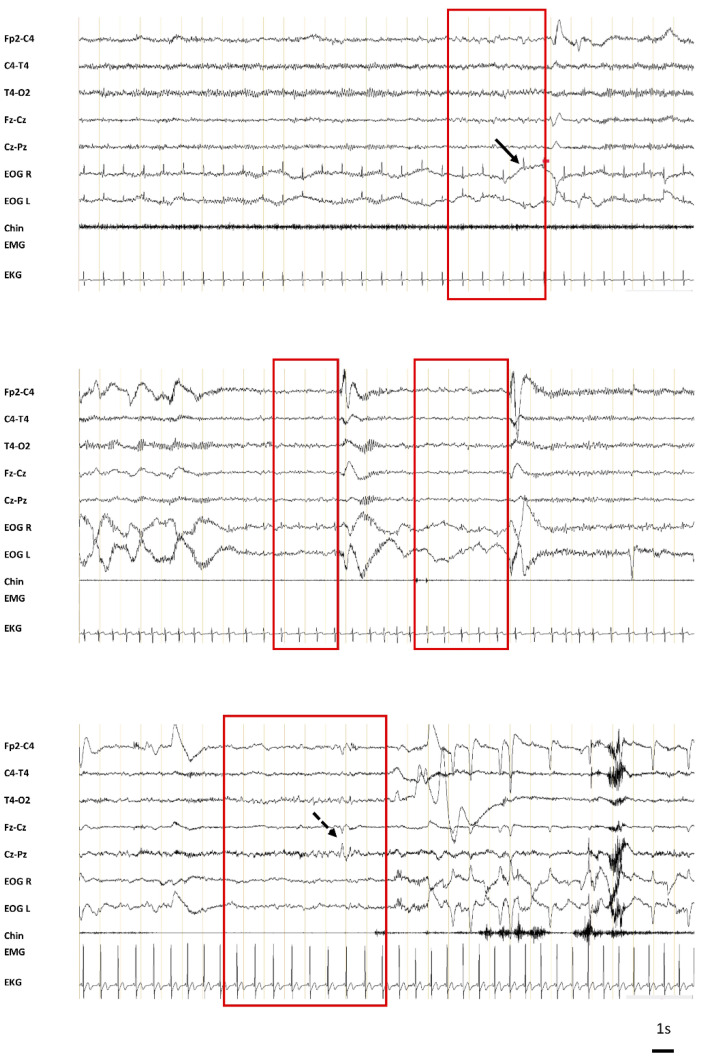
Examples of microsleeps. The microsleeps (MSs) are highlighted in the red boxes. They mostly present as a disappearance of alpha rhythm (eyes closed) with or without slow theta waves. The duration of the MS is clearly less than half of the epoch, and therefore this epoch is not allowed to be scored as sleep. The black arrows point to slow eye movements typical of the N1 stage and the dotted black arrow points to a vertex wave (EEG channels: bipolar montage (Fp2-C4; T4-T4; T4-O2, Fz-Cz, Cz-Pz); EOG: electro-oculogramm right (R) and left (L); EMG: electromyogram; EKG: electrocardiogram).

A total of 526 MSs were recorded during the four tests in the 98 patients, and at least 1 MS was recorded in 61 patients. Among these MSs, 72% were scored by both scorers 1 and 2, and the remaining 28% were scored by scorer 1 and scorer 3 (7%), or scorer 2 and scorer 3 (21%). Examples of MSs are shown in Figure 1. They mostly presented as theta waves occurring alone (85.7%) or associated with slow eye movements (12.5%). In some cases, sleep features such as vertex waves (1.2%), K-complexes (0.2%), or delta waves (0.4%) were observed.

**Figure 2:**
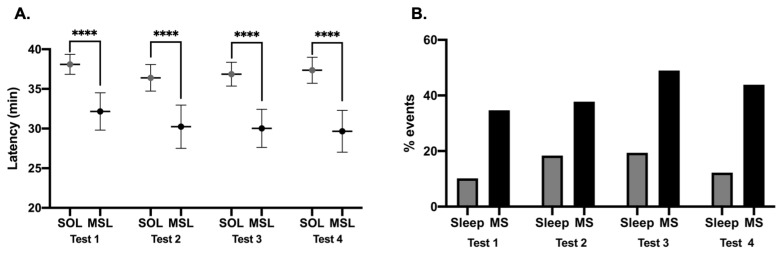
Effect of test time on microsleep and sleep episodes. Test 1 is performed at 9:00 a.m., Test 2 at 11:00 a.m., Test 3 at 1:00 p.m., and Test 4 at 3:00 p.m. (**A**) Mean microsleep latency (MSL) and sleep onset latency (SOL). Latencies are presented as mean with 95 CI. The MSL (black dot) is always significantly shorter than the SOL (grey dot). No significant difference between the 4 tests is observed. (**B**) Proportion of patients who fell asleep (MS or sleep) for each test. No significant difference between the 4 tests is observed. **** *p* < 0.05.

The number of MSs was significantly higher during tests with (N = 59) sleep than without (N = 333) sleep (median 2.0 (3.0–4.0) versus 0 (0.0–0.0), *p* < 0.0001). However, in almost a quarter of the tests (N = 92 in 26 patients), at least one MS without falling asleep was recorded. The maximum number of MSs during a test was 16, with a median of 1 for the first two tests and of 2 for the last two tests in patients with MSs. The median (Q1–Q3) total number of MSs over the four tests was 8 (3–12). The average duration of MSs was 5.9 s (± 2.8). When sleep was preceded by more than 1 MS (N = 45 tests), the duration of the last MS before sleep onset was significantly longer than that of the first MS (median 7.0 s (5.0–8.5) versus 4.0 s (3.5–7), *p* < 0.001). In addition, MSs recorded in tests during which the patient fell asleep (N = 324 MS in 55 tests) were significantly longer than those recorded in tests without sleep (N = 202 MS in 106 tests) (6.3 ± 3.2 s versus 5.7 ± 2.5 s, *p* = 0.023). Changes in SOL and MSL according to the timing of the test are presented in Figure 2A. A repeated measures two-way ANOVA revealed a significant effect of the latency used (SOL versus MSL: F(1, 97) = 81.26, *p* < 0.0001) but not of the test timing, and no interaction between these two factors. Post-hoc analyses showed that SOL was consistently higher than MSL: test 1, mean difference = 5.94 min with 95% CI (3.17–8.71), *p* < 0.0001; test 2, mean difference = 6.16 min with 95% CI (3.39–8.93), *p* < 0.0001; test 3, mean difference = 6.83 min with 95% CI (4.06–9.60), *p* < 0.0001; test 4, mean difference = 7.69 min with 95% CI (4.92–10.46), *p* < 0.0001. The proportion of patients with at least one MS or with sleep did not significantly differ between the four tests either, despite a trend towards higher values at test 3 (performed at 1:00 p.m.) (Figure 2B).

### 2.4. MWT Results

**Figure 3:**
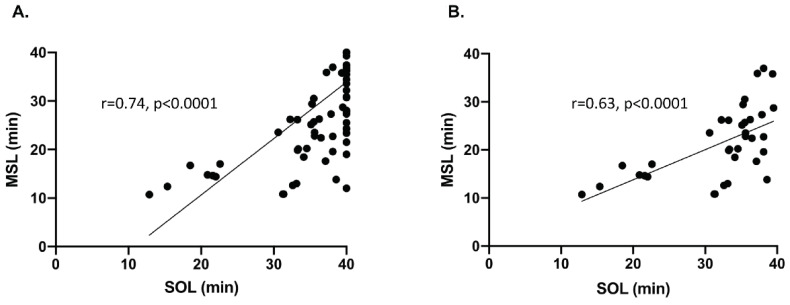
Correlation between the microsleep latency (MSL) and the sleep onset latency (SOL). Each point represents a patient defined by his mean MSL and his mean SOL across the 4 tests. (**A**) All MWT with at least one MS are taken into account (N = 61), even those with no sleep (mean SOL = 40 min). A significant positive correlation is observed between SOL and MSL (r = 0.74, 95% CI (0.63; 0.82), *p* < 0.0001). (**B**) The same representation excluding tests with no sleep (N = 35). The positive correlation remains significant (r = 0.63, 95% CI (0.37; 0.80), *p* < 0.0001).

The mean SOL calculated over the four tests in was 37.2 (±5.7) min (median 40.0, min 12.9 max 40.0), with 16 patients exhibiting with a mean SOL below 34 min and only three patients with a mean SOL below 20 min (medico-legal threshold in France). One MS was observed during at least one of the four tests in 62.2% of the patients. The mean MSL during the four tests was 30.6 (±9.8) min (median 34.0, min 10.7 max 40.0), with 49 patients exhibiting with a mean MSL below 34 min and 15 patients with a mean MSL below 20 min. The mean MSL among patients who had MSs was 24.8 (±8.4 min). The MSL was significantly positively correlated with the SOL (r = 0.74, 95% CI (0.63; 0.82), *p* < 0.0001) (Figure 3).

In all cases but three, when sleep was observed during a test, it was preceded by one or more MSs, on average by 3.4 (± 2.9) MSs. Differences between the three groups of patients (defined according to SOL and MSL) were found for age, Pichot scale, and sleep efficiency, but not for other variables (gender, diagnosis, other subjective questionnaires, sleep debt, other polysomnography parameters, car accident history). Data for these three groups are presented in Table 3.

Considering the whole population, no significant correlation was observed between the MSL or the SOL and demographic (age, sex, BMI), clinical (ESS, Pichot scale, BDI) and PSG variables (sleep efficiency, arousal index, AHI, time spent with SpO_2_ < 90%), except for a weak negative correlation between the SOL and ODSI score (r = −0.27, *p* = 0.05).

**Figure 4:**
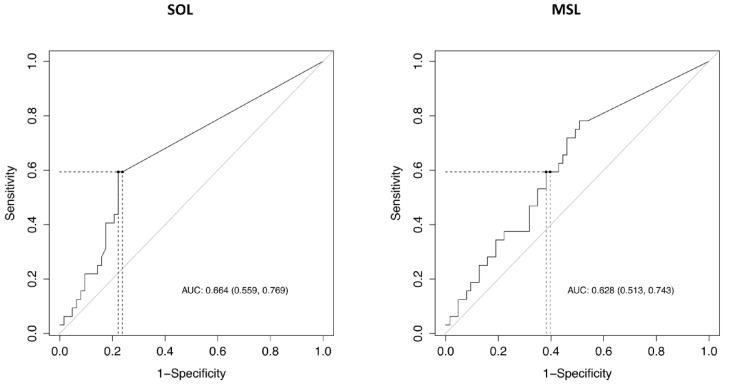
Diagnostic value of Maintenance Wakefulness Test (MWT) for excessive daytime sleepiness according to whether or not MSs are included in the interpretation.

The ROC curves for the diagnosis of excessive daytime sleepiness as assessed by the ESS are presented in Figure 4 and show a similar performance for the MWT whether the test is interpreted with or without the MSs. The areas under the ROC curve for the prediction of sleepiness were 0.66 95% CI (0.56, 0.77) for SOL and 0.63 95% CI (0.51, 0.74) for MSL. Sensitivities, specificities, and positive and negative predictive values according to the selected thresholds are detailed in Table 4. Overall, high specificities were obtained for SOL < 34 min and for MSL < 20 min.

## 3. Discussion

In this study, we report for the first time the results of MS assessment during the MWT performed as a routine evaluation in a large cohort of patients treated for sleep disorders. We found: (i) that MSs are frequent during MWT, even in a treated population, since 62.2% of the patients presented at least one MS; (ii) significant correlations between the latency of the first MS, the number and duration of MSs on the one hand, and the presence of sleep and sleep onset latency on the other hand, suggesting that MSs are good markers of drowsiness prior to falling asleep; and (iii) a lack of correlation between the latency of the first MS and subjective sleepiness as assessed by scales and questionnaires, and an overall poor diagnostic performance of MSs (as well as SOL) to assess subjective sleepiness.

### 3.1. MSs as Physiological Phenomena While Falling Asleep

Sleepiness involves multiple dimensions, including a subjective feeling of drowsiness, objective behavioral and cognitive (attentional) impairment or “lapses” [35], and changes in various neurophysiological parameters (brain activity, eye movements, muscle tone, etc.). These latter changes observed in PSG recordings have given rise to the first definition of MSs, characterized by the appearance of slow rhythms (delta, theta), sometimes associated with other features usually observed during the light stages of NREM sleep, such as vertex waves or slow eye movements [25]. In our study, using this PSG definition of MSs, we found that most MSs presented as short fragments of N1 stage, whose short duration (5.9 s on average per MS, i.e., less than 15 s/epoch) and low recurrence prevented us from scoring the epoch as a sleep epoch. These MSs were observed in almost two-thirds of patients during at least one of the four tests. Interestingly, sleep onset was almost always preceded by at least one MS, and the latency of the first MS, the duration of MSs, and the number of MSs were correlated with the presence of sleep. These results suggest that MSs are physiological events preceding sleep onset, although their prevalence may have been enhanced in our study by the particular context of the MWT, during which the patients had to resist falling asleep in a passive environment.

Our findings echo a recent work about MSs during MWTs performed on 76 patients with excessive daytime sleepiness, without details of the underlying sleep disorder or treatment status [32]. In this study, the authors reported the serial occurrence of MSs (from one to seven maximum, with an average of three MSs) before sleep, which is corroborated by our work. A strong correlation between the sleep onset latency and the latency of the first MS was also reported, in line with our findings. In 112 treatment-naïve OSA patients, Morrone et al. reported MSs in 71% of patients and found that MS detection improved the ability of the MWT to discriminate sleepy from sleep-resistant patients as defined by the MWT [33]. However, the mean MS latency (8.4 +/− 6.8 min) was much lower than that observed in our work (30.6 +/− 9.8 min), probably because these patients had severe untreated OSA. The context in which our patients were tested (before resuming work or driving) may have also influenced the results as most patients were highly motivated to stay awake.

These studies and our findings are in line with basic science work on animals and humans, arguing for progressive and fluid, rather than clear-cut, transitions between vigilance states, and suggesting that falling asleep does not affect the whole brain in a global way [36]. Therefore, intra-cerebral recordings have allowed the observation of an asynchrony in sleep onset between the thalamus, limbic structures, and neocortical area, suggesting that falling asleep is a gradual process [37,38]. Intrusion of sleep during wakefulness, defined as global or local transient slow waves associated with a decrease in the neuronal firing rate, has also been observed in sleep deprivation conditions and has been correlated with performance impairment or “cognitive lapses” [39,40]. The low spatial resolution of scalp EEG performed for PSG purposes did not allow us to look for local specificities in MS episodes; however, these MSs may constitute markers of sleepiness at a macroscale level, reflecting the intrusion of brief periods of sleep during wakefulness before the installation of stable sleep.

### 3.2. MS in the Context of MWT

Interestingly, we found no correlation between MSL and the various clinical parameters used to assess subjective sleepiness, nor did we find any improvement in the MWT performance for the diagnosis of excessive daytime sleepiness when considering MSL rather than SOL. Such discrepancies between patient-reported and objectively measured sleepiness (with either the MSLT or MWT) have already been reported [10,31,33]. Thus, the poor correlation between sleep latency and the ESS was not significantly improved by using MSL rather than SOL in patients with sleep disorders [31], even if discordant findings have been reported by another team [30]. In the context of the MWT, no significant difference in the ESS score was found between three groups of non-treated OSA patients defined by the sleep latency [33]. Subjective sleepiness perception may depend on the context of the evaluation, with poorer spontaneously perceived sleepiness at the time of MS episodes (defined as >3 s of theta activity) by healthy subjects during the MWT than during a simulated driving condition [10].

This divergence between electrophysiological markers of sleepiness and subjective scales could be due to a perceptual bias, to a (voluntary) reporting bias, or to the fact that sleepiness is a multidimensional phenomenon whose subjective scales and objective tests measure different aspects. A subjective perception of sleepiness may be impaired by the sleepiness itself; insight about sleepiness appears to be particularly disturbed among OSA patients, with a strong tendency to minimize symptoms prior to treatment and, ultimately, retrospective reporting of pre-treatment sleepiness [41]. The absence of correlation (or weak correlations) between the ESS and MSLT results has also been reported in several populations of patients with sleep disorders [14,42,43,44,45]. To note, MSLTs are usually performed to diagnose excessive daytime sleepiness. Thus, the potential intentional reporting bias in the subjective self-assessment of their drowsiness is thought to be limited. On the contrary, MWTs are mainly used to explore vigilance in a professional or driving context, which may be responsible for a declarative bias. In our study, the fact that the decision to resume work depended on the MWT results in many patients may have influenced the self-evaluation of sleepiness in some of them.

We observed that sleep-related breathing disorders, which were the main cause of vigilance disorders in our patients, were overall well controlled, with 66% of patients having no or mild OSA. The ESS was normal in 66% of patients and the SOL-based MWT was also normal in the majority of patients (83.7% of patients). We did not observe any correlation between MSL and PSG parameters, including sleep quality or AHI. Two-thirds of patients had MSs, including those who did not fall asleep, did not report subjective sleepiness, and had a normal pre-MWT PSG. This observation raises questions about the physiological meaning of MS, about their actual behavioral impact, especially in driving situations, and about the fact that the MWT protocol may not be the most relevant test to assess driving ability.

### 3.3. Correlation between MS and Subjective Sleepiness

The issue of the MWT, beyond the quantification of sleepiness, is dominated by the objective of predicting a loss of performance related to sleepiness, especially at the wheel. Several studies in the field have used the concept of “lapses” to refer to momentary interruptions in a subject’s reactivity that cause behavioral errors [46]. A strong correlation between the crash risk in a driving simulation paradigm and the incidence of MSs has been reported in a healthy population [47]. Other authors have reported that MSs in healthy subjects occurred more frequently during monotonous, rather than complex, tasks, with a higher cognitive load increasing the likelihood of attention loss and behavioral errors rather than MSs [48]. In OSA patients, an association between EEG-defined attention lapses (disappearance of alpha rhythm or the appearance of theta rhythm for at least 3 s) and errors on a simulated driving test was also reported [27]. Such findings were confirmed in another group of OSA patient who underwent a driving simulator task with synchronous EEG recording; in this study, the patients showed significant deterioration in vehicle control during MSs compared to driving performance in the absence of MSs, and the degree of performance decrement correlated with the MS duration [26]. These findings suggest an association between MSs and behavioral/attentional impairment, and therefore a risk of a car crash in patients with MSs while driving. However, a recent study showed that, in spite of a correlation between the presence of MSs and errors on a driving simulator at the same time, no significant correlation could be found between MSs recorded during the MWT and driving performance [49]. In line with this finding, another team reported that the MWT results poorly correlated with driving performance during a 2-hour task irrespective of sleep latency cutoff or added alpha/microsleep latency data [50]. Thus, it seems that, if MSs are associated with impaired performance at the time of their occurrence (e.g., in the driving simulator), their presence during the MWT poorly predicts impaired driving performance. This could suggest that the MWT may not constitute an optimal test for assessing driving ability; they are performed in a situation of extreme passivity, not encountered while driving, and may therefore be too “severe” as compared to driving simulation tasks. However, these latter paradigms are currently not validated to allow driving in the context of sleep disorders, and do not fully avoid the pitfall of being a virtual situation far from real driving.

### 3.4. Strengths and Limitations

The strengths of our study are the large size of our population, the presence of subjective scales at the time of the MWT, and a full night PSG the night before the MWT. In addition, our patients are representative of patients that are usually explored, after treatment, in the context of the MWT to assess residual sleepiness before resuming driving. However, our work has several limitations. First, as discussed above, a declaration bias in subjective sleepiness assessment cannot be excluded given the implications of the MWT in our patients. Second, the sleepiness evaluation was performed with the ESS which does not provide an instantaneous measure of sleepiness, unlike the Karolinska Sleepiness Scale [51]. Third, analyses were performed on a bipolar montage which is not conventional according to the American Academy of Sleep Medicine (AASM) procedure and scoring manuals [52]. However, we do not believe that this would limit the generalizability of our findings as there is no evidence that the montage significantly influences visual scoring [53,54]. Fourth, the absence of video data did not allow us to analyze behavioral correlates (facial movements, etc.), which could have been interesting in this context. A recent study used not only EEG and EOG, but also visual analysis of face videography (eyelid position) to detect MSs [32]. To note, the duration criteria was different in this study (from 1 to 15 s) and the authors showed that 40% of MSs lasted between 1 and 3 s. These results suggest that the actual number of MSs may be higher than that observed in our study with the 3–15 s duration criterion. However, the visual scoring of the MS is complex, with variability from one reader to another. The 3-s threshold used in our work, as well as the triple scoring, allowed more specific detection. In clinical practice, there are several barriers to the use of MSL in MWT scoring, such as the lack of well-defined widely recognized scoring criteria and the fact that this scoring is time-consuming. The BERN continuous and high-resolution wake-sleep scoring criteria have recently been proposed to identify an MSE (microsleep episode) and MSEc (microsleep episode candidate) according to their duration, to video analysis data, and to EEG and EOG recordings [32]. An automatic detection of MS events with feature-based machine learning demonstrated good performances as compared to visual detection and may facilitate the systematic search for MSs in the future, should the relevance of their use in clinical practice be established [55].

## 4. Materials and Methods

### 4.1. Study Population

All consecutive adult patients referred to the Center for Sleep Medicine and Respiratory Diseases for a full night PSG followed by an MWT in the context of a residual sleepiness assessment between September 2017 and January 2019 were retrospectively screened to be included in the study. The exclusion criteria for the study were: the presence of encephalopathy or epilepsy with an abnormal EEG likely to interfere with the identification of MS (N = 0), failure to perform the four tests correctly for 40 min as recommended by the American Academy of Sleep Medicine (AASM) (N = 1), and patient’s decline to participate in the study (N = 1). All patients gave informed consent for the use of their data for research purposes. The study was approved by the Hospices Civils de Lyon Ethical Committee on 13 January 2020 (No. 20-04) and registered on the Clinical Trials website (NCT04559269).

### 4.2. Clinical Data

The following clinical data were reviewed in patients’ medical files: age, sex, medical history, current treatments, body mass index (BMI), and wake-promoting substances (coffee, tea, etc.), as well as tobacco and alcohol consumption. Their sleep habits (sleep-wake schedules on working days and on days off) were collected. As part of the routine diagnosis evaluation, patients were asked to fill out the Epworth Sleepiness Scale [56], the Pichot Fatigue Scale [57], the Beck Depression Inventory [58], and the ODSI Sleepiness Scale [45,59]. The morning following the PSG, patients filled out the St Mary’s Hospital Morning Rating Scale [60]. The reason why the tests were performed (professional driver, safety job, before driver’s licensing, patient’s wishes) was also noted.

### 4.3. Recordings

#### 4.3.1. Recording Procedure

The night before the MWT, full PSG was conducted in the Center for Sleep Medicine and Respiratory Diseases. Patients arrived in the late afternoon and underwent instrumentation for the electrodes and sensors required for PSG. The following signals were recorded: electroencephalogram (Fp2-C4; C4-T4; T4-O2; Fp1-C3; C3-T3; T4-O2; Fz-Cz; Cz-Pz), EOG (mastoid reference), chin and tibialis electromyogram (EMG), EKG, nasal airflow (nasal pressure and thermistor), pulse oxymetry, microphone, and respiratory efforts (thoracic and abdominal). For OSA patients, PSG was performed with continuous positive air pressure (C-PAP) or oral appliance therapy (OAT). Patients with wake-promoting medications also took their treatments at the usual times. Bedtime was free but patients were informed that they would be woken up at 7:00 a.m. for the MWT protocol and that they should have slept at least 6 h before the MWT.

A standard MWT protocol was administered to patients according to the American Academy of Sleep Medicine (AASM) guidelines [17]. The first test began 1.5 h to 3 h after termination of the nocturnal recording, at 9:00 a.m. in most cases. Four 40-minute tests were performed at two-hour intervals. Between tests, patients were free to move around, but the consumption of stimulants was monitored and they were not allowed to take naps. Each test was interrupted if three epochs of N1 stage or one page of any other sleep stage occurred.

#### 4.3.2. Recording Analysis

- Polysomnography: usual sleep parameters were collected—total sleep time (min), sleep efficiency (%), sleep onset latency (SOL), time spent in N3 stage (min, %), time spent in REM sleep (min, %), apnea-hypopnea index (n/h), arousal index (n/h), and time spent with SaO2 < 90% (min).
- MWT: for each patient, the four tests were scored by two independent scorers (scorer 1: LPD and scorer 2: TP) with the intervention of a third scorer to reach a consensus in case of disagreement (scorer 3: HB). The scoring included:
  - SOL defined as the time until the first epoch of whatever sleep stage, i.e., at least 15 s of cumulative sleep on a 30 s epoch. If the patient did not fall asleep, SOL was considered to be 40 min.
  - The occurrence of one or more MSs during the entire test (in case the patient did not fall asleep) or until sleep onset. MSs were defined by the appearance of theta or delta waves with a disappearance of alpha in the absence of eye opening, sometimes associated with slow eye movements, lasting from 3 to 15 s. The latency, duration, number, and EEG description of the MSs were collected. In the absence of MS, the sleep latency considered for the test was SOL (or 40 min in the absence of sleep).

### 4.4. Statistical Analysis

Continuous variables were described by the median and first and third quartiles (Q1–Q3) or mean (±SD) according to their distribution; categorical variables were described by the frequency and percentage of each modality (excluding missing data from percentages).

Patients were classified into three groups according to the mean SOL and to the mean MSL—group 1: normal SOL and MSL (both >33 min); group 2: mean SOL >33 min but mean MSL ≤33 min; and group 3: mean SOL ≤33 min. The 33-min value was chosen given that the MWTs were performed for professional driving purposes in most patients and that this latency had been correlated with normal driving performance [24].

Comparisons of different clinical and paraclinical variables between the three groups defined above were performed using the Kruskal–Wallis test. Comparison of SOL and MSL between the four tests of each MWT session was performed with a repeated-measures two-way ANOVA. The duration of MSs in the same patient during the same test (first versus last MS) were compared with the Wilcoxon test for paired data and between tests (with or without falling asleep) with the Mann–Whitney test. The number of MSs occurring in tests with and without sleep was compared with the Mann–Whitney test. The duration of the MSs for the tests with and without falling asleep were compared with a t-test. The correlation between SOL and MSL was assessed with the Spearman correlation test with its 95% confidence interval (95% CI). The sensitivity and specificity of the different latencies for diagnosing excessive sleepiness (Epworth Sleepiness Scale >10) were calculated for different values of mean SOL and mean MSL. The diagnostic value of the two latencies was evaluated by the area under the ROC (receiver operating characteristic) curve and its 95% CI.

A value of *p* < 0.05 was considered as statistically significant. All analyses were performed using R (R Foundation for Statistical Computing, Vienna, Austria).

## 5. Conclusions

Microsleeps are commonly recorded during MWTs and may represent physiological markers of the wake-to-sleep transition with few patients reaching sleep without going through several MSs. However, MSs are also observed in patients usually considered as “non-sleepy” based on normal MWT sleep latency, normal subjective vigilance scales, and effective OSA treatment, as evidenced by PSG. Although the behavioral correlates of MS in terms of attentional lapses have been reported in many studies, the value of their detection during MWTs for predicting car accidents remains to be explored.

## Figures and Tables

**Table 1:** Demographic and clinical characteristics of the patients.

	**Mean ± SD (min-max) or n (%)**
**Age**	
Gender (%F) Diagnosis (OSA, OSA + narcolepsy, OSA + hypersomnia, narcolepsy) %	45.3 ± 10.8 (19.0–71.0) 17% 86.7%, 3.1%, 1%, 9.2%
BMI (kg/m^2^) (N = 98)	30.6 ± 6.7 (18.8–57.8)
**Medical past**	
Tobacco (N = 93)	26 (28%)
Hypertension (N = 95)	23 (24.2%)
Stroke (N = 93)	2 (2.1%)
Myocardial infarction (N = 94)	1 (1.1%)
Lower extremity arterial disease (N = 94)	0 (0%)
Depression (N = 93)	20 (21.5%)
Daily wake-promoting drinks consumption (coffee (N = 92), tea (N = 93), others (N = 93))	76 (82.6%), 38 (40.9%), 28 (31.1%)
**Sleep duration**	
Sleep duration on work days (h) (N = 80)	7.2 ± 1.2 (4.0–10.5)
Sleep duration on holidays (h) (N = 91)	8.9 ± 1.8 (6.0–17.0)
Sleep debt (h) (N = 78)	1.9 ± 2.0 (1.0–10.7)
**Questionnaires**	
Epworth Sleepiness Scale (N = 95)	8.0 ± 4.9 (0.0–20.0)
Pichot fatigue scale (N = 89)	8.3 ± 7.3 (0.0–29.0)
ODSI (N = 80)	6.3 ± 5.9 (0.0–22.0)
BDI (N = 85)	6.0 ± 6.3 (0.0–35.0)

N: number of patients who answered the question; OSA: obstructive sleep apnea; BMI: body mass index; ODSI: Interview-based Diurnal Sleepiness Inventory; BDI: Beck Depression Inventory. The sleep debt is calculated as the difference between sleep duration on holidays and sleep duration on workdays.

**Table 2:** Sleep parameters on polysomnography the night before the Maintenance Wakefulness Test.

	Median (Q1–Q3)
Total sleep time (min)	410.0 (366.0–453.8)
Sleep latency (min)	22.4 (12.3–35.9)
Wake after sleep onset (min)	47.0 (26.3–84.3)
Sleep efficiency (%)	81.4 (70.4–87.8)
Time spent in N3 sleep (min and %)	87.0 (64.3–113.5) 21.6 (16.5–30.6)
Time spent in REM sleep (min and %)	86.0 (60.3–111.0) 20.0 (16.2–24.8)
Arousal index (n/h)	21.4 (15.0–28.6)
AHI (n/h)	8.2 (3.1–18.0)
AHI + RERA (n/h)	11.8 (4.9–22.0)
Time spent with SaO2 < 90% (s)	0.00 (0.0–27.8)

Values are presented as median with 25% and 75% quartiles. AHI: apnea-hypopnea index; RERA: respiratory effort-related arousal.

**Table 3:** Comparisons between the three groups of patients (continuous data are presented in medians (Q1–Q3).

	Group 1 (N = 51) Normal SOL and MSL (> 33 min)	Group 2 (N = 31) SOL > 33 min but MSL ≤ 33 min	Group 3 (N = 16) SOL ≤ 33 min	*p*-Value
Age (years)	48.0 (40.0–53.8)	44.0 (29.8–51.0)	50.5 (43.8–56.3)	0.04
Gender (% male)	90.0%	75.0%	75.0%	0.14
BMI (kg/m^2^)	30.3 (26.3–33.7)	28.9 (25.9–32.6)	30.2 (27.5–37.3)	0.57
Epworth Sleepiness Scale	7.0 (4.0–10.0)	7.0 (4.0–13.0)	9.0 (7.0–14.0)	0.13
% abnormal	24.0%	40.6%	43.8%	0.18
Pichot Scale	5.5 (2.0–12.0)	6.00 (2.0–11.0)	11.00 (5.0–21.3)	0.18
% abnormal	4.0%	3.1%	12.5%	0.02
ODSI	4.0 (2.0–10.0)	3.0 (2.0–8.8)	6.0 (2.0–12.0)	0.67
% abnormal	50.0%	53.1%	62.5%	0.86
BDI	4.0 (1.0–9.0)	5.5 (1.0–8.0)	7.0 (1.0–10.0)	0.99
% abnormal	4.0%	0.0%	0.0%	0.65
Diagnosis (%OSA, OSA + narcolepsy OSA + hypersomnia, narcolepsy)	94.0%, 2.0%, 0.0%, 4.0%	77.4%, 3.2%, 3.2%, 16.1%	81.2%, 6.3%, 0.0%, 12.5%	0.19
Total sleep time (min)	391.5 (350.8–432.3)	432.5 (392.0–461.5)	407.0 (372.8–458.5)	0.13
Sleep efficiency (%)	77.2 (68.9–85.5)	85.6 (78.34–93.5)	80.3 (71.1–86.7)	0.03
Wake after sleep onset (min)	55.0 (30.0–55.0)	34.5 (18.5–34.5)	47.50 (31.8–47.5)	0.11
Time spent in N3 sleep (min)	89.0 (64.3–108.5)	94.0 (81.8–135.0)	81.0 (66.3–110.3)	0.20
Time spent in REM sleep (min)	79.5 (58.5–111.0)	95.0 (79.8–110.8)	78.5 (63.5–101.0)	0.29
Arousal index (n/h)	22.6 (16.5–28.3)	20.5 (11.4–26.6)	22.1 (17.9–34.9)	0.4
AHI (n/h)	7.8 (2.5–17.7)	8.2 (4.2–17.4)	13.0 (3.2–29.2)	0.45
AHI + RERA (n/h)	12.3 (4.4–20.3)	8.6 (5.1–20.9)	14.5 (5.3–31.1)	0.5
Time spent with SaO2 <90% (s)	0.00 (0.00–19.5)	0.00 (0.0–20.0)	0.00 (0.0–39.97)	0.84
Sleep debt (h)	1.5 (0.9–2.0)	2.0 (1.0–3.0)	1.0 (0.0–2.6)	0.37
Car accident in the last two years	18%	12.5%	37.5%	0.1

**Table 4:** Diagnostic values of sleep onset latency (SOL) and microsleep latency (MSL) for identifying excessive daytime sleepiness as assessed by the Epworth Sleepiness Scale.

Test	Sensitivity	Specificity	Positive Predictive Value	Negative Predictive Value
SOL < 34	0.22	0.86	0.44	0.68
SOL < 20	0.06	0.98	0.67	0.67
SOL < 12	0.03	1.00	1.00	0.67
MSL < 34	0.66	0.55	0.43	0.76
MSL < 20	0.25	0.84	0.44	0.69
MSL < 12	0.06	0.97	0.50	0.67

These figures show two ROC curves for the prediction of subjective sleepiness (Epworth score > 10) according to the mean SOL and the mean MSL of MWT. Performances are close for the two latencies (0.66 95% CI (0.56, 0.77) for SOL and 0.63 95% CI (0.51, 0.74) for MSL).

## Data Availability

The data that support the findings of this study are available from the corresponding author upon reasonable request.
